# The effect of peer modelling and discussing modelled feedback principles on medical students’ feedback skills: a quasi-experimental study

**DOI:** 10.1186/s12909-021-02755-z

**Published:** 2021-06-08

**Authors:** Floris M. van Blankenstein, John F. O’Sullivan, Nadira Saab, Paul Steendijk

**Affiliations:** 1 Center for Innovation in Medical Education, Leiden University Medical Center, P.O. Box 9600, 2300 RC Leiden, the Netherlands; 2 Leiden University Graduate School of Teaching, Leiden University, Leiden, the Netherlands; 3 Department of Cardiology, Leiden University Medical Center, P.O. Box 9600, 2300 RC Leiden, the Netherlands

**Keywords:** medical students, undergraduate education, peer feedback, peer review, observational learning, cognitive elaboration, peer modelling, modelling examples

## Abstract

**Background:**

Teaching is an important professional skill for physicians and providing feedback is an important part of teaching. Medical students can practice their feedback skills by giving each other peer feedback. Therefore, we developed a peer feedback training in which students observed a peer that modelled the use of good feedback principles. Students then elaborated on the modelled feedback principles through peer discussion. This combination of peer modelling and discussing the modelled feedback principles was expected to enhance emulation of the feedback principles compared to (1) only peer modelling and (2) discussing the feedback principles without previous modelling.

**Methods:**

In a quasi-experimental study design, 141 medical students were assigned randomly to three training conditions: peer modelling plus discussion (MD), non-peer modelled example (NM) or peer modelling without discussion (M). Before and after the training, they commented on papers written by peers. These comments served as a pre- and a post-measure of peer feedback. The comments were coded into different functions and aspects of the peer feedback. Non-parametrical Kruskall-Wallis tests were used to check for pre- and post-measure between-group differences in the functions and aspects.

**Results:**

Before the training, there were no significant between-group differences in feedback functions and aspects. After the training, the MD-condition gave significantly more positive peer feedback than the NM-condition. However, no other functions or aspects were significantly different between the three conditions, mainly because the within-group interquartile ranges were large.

**Conclusions:**

The large interquartile ranges suggest that students differed substantially in the effort placed into giving peer feedback. Therefore, additional incentives may be needed to motivate students to give good feedback. Teachers could emphasise the utility value of peer feedback as an important professional skill and the importance of academic altruism and professional accountability in the peer feedback process. Such incentives may convince more students to put more effort into giving peer feedback.

## Background

Teaching is regarded internationally as an important physician skill [1, 2]. Medical students can learn this skill by teaching their peers [3–6], which can be operationalised as *near-peer teaching* when the teaching student is more advanced than the learning student, or *same-level* teaching when students have no developmental differences [7, 8]. One way of *same-level* peer teaching is giving peer feedback, which has been implemented in medical education to assess e.g. professional behaviour [9], teaching skills [10] and communication skills [11]. Giving peer feedback allows students to develop professional teaching skills, like evaluating performance, justifying evaluations and helping others improve [12–14]. It may also improve their own learning performance [15–18]. Therefore, peer feedback can be used to prepare medical students for their future teaching role as a physician.

However, research shows that students need feedback training in order to give good peer feedback [14, 19–21]. Several training methods have been developed for this purpose [22–26], but a method that has not been studied frequently is peer modelling. Modelling in general means that a person (e.g. a teacher) demonstrates how to perform a certain task or behaviour [27]. In the case of *peer modelling*, these persons are fellow students. Peer modelling has been researched for various learning tasks [28–30] and has shown to be effective for improving e.g. writing skills [31, 32]. However, to our knowledge only one study investigated the effect of peer modelling on giving peer feedback [33]. In that experimental study, students either observed peer models who demonstrated a text review strategy or practiced the same review strategy at once. Students then emulated the review strategy either individually or in pairs. After observing the peer models, students who emulated the review strategy in pairs used more features of the review strategy in their own peer reviews than students who emulated the review strategy individually. In contrast, after practicing the review strategy right away, students who emulated the review strategy in pairs used *fewer* aspects of the review strategy than students who emulated the strategy individually. In other words, there was a significant interaction effect between type of instruction (peer modelling or practice) and follow-up activity (emulation in pairs or alone). This interaction effect suggested that observing peer modelling was only effective when followed by emulation in pairs.

Theoretically, this effect can be explained as cognitive elaboration that occurs when students discuss new knowledge with fellow students [34, 35]. Peer discussion is a powerful form of active learning that can lead to co-construction of knowledge, the formation of new ideas [36, 37] and the development of elaborate mental models [38]. In the case of peer modelling, students may elaborate more deeply on what peer models demonstrate when they discuss the modelled performance with each other. As a consequence, they may emulate more features of the modelled performance.

In sum, medical students can practice future teaching skills by giving peer feedback. Modelling good feedback principles may be an effective method to teach students how to give peer feedback, especially when modelling is followed by peer discussion in order to elaborate on the modelled feedback principles. However, this hypothesis has not been tested yet in medical education.

Therefore, we investigated the effect of peer modelling followed by peer discussion of the modelled feedback principles on students’ emulation of the feedback principles. We expected that peer discussion would increase elaboration on the feedback principles and emulation of these principles. As a consequence, we hypothesised that peer modelling plus discussion would lead to more emulation of the feedback principles than either peer modelling alone or peer discussion of the feedback principles without previous modelling of these principles.

## Methods

### Design and setting

This study used a quasi-experimental, between-groups, pre-/post-measure design with three conditions: peer modelling plus discussion (MD), non-peer modelled example (ED), or peer modelling without discussion (M). The pre- and post-measures consisted of annotated peer feedback comments. The study took place in a Dutch medical school while students were writing their bachelor thesis (in the Netherlands, medical school starts at the bachelor level, when students are approximately 18 years old). In this particular medical school, students give peer feedback on multiple occasions throughout their undergraduate bachelor curriculum. For instance, in year 1 they role play consultations in the role of patient, doctor or observer and give peer feedback to the doctor. In addition, they peer review academic writing assignments in their first and second year of study. Therefore, peer feedback is implemented consistently throughout the curriculum.

This study took place in the third (also last) year of the bachelor program. The bachelor thesis was a ‘Critical Appraisal of a Topic’ (CAT): a structured synthesis of research literature based on a clinical question, including a literature search strategy, a critical appraisal of the literature and an explanation of the clinical application of the results [39]. Students wrote their CAT-paper as part of a research project that lasted several months. They were supervised by PhD students and communication teachers who taught academic writing. The communication teachers also taught sessions in which students learned how to peer review each other’s papers and the training took place during these sessions. In these sessions, they explained the importance of peer feedback to students by emphasising that peer feedback is an important academic and professional skill. Moreover, it was discussed with students that the process of providing feedback would develop their own abilities as academic communicators [18]. Students were required to peer review CAT-papers of other students before and after these sessions.

### Participants

 All students who started their bachelor thesis were invited to participate in the study through an informed consent letter which they could sign if they wanted to participate. Thus, students who wanted to participate gave written informed consent. 245 students (mean age 21.0 years, SD = 1.4) signed this letter and within this group, complete data of 141 students was obtained.

### Materials

One of the communication teachers selected a CAT-paper that needed improvement of a student from the previous year. The teacher wrote annotated feedback comments in the second chapter of this thesis. Informed by literature on good feedback principles [25, 40–42], these comments contained *evaluations* of performance (i.e. explicit or implicit comments on the quality of the text), *explanations* for these evaluations and *suggestions for improvement*. For instance, one of the comments was: ‘It is clear that it’s the final bit of information for the section – but I think that you could give a little more information, or tie it all together somehow. Maybe summarise a few main points?’ This comment contained a positive evaluation that was explained, and two suggestions for revision. Table 1 contains some more examples of how the feedback principles were modelled. Specific attention was paid to including positive evaluations in the feedback, because the communication teachers noted that students often forgot to give positive peer feedback.

**Table 1:** Examples of modelled feedback principles

Principle	Example 1	Example 2
Evaluation	This is a little unclear to me	And, I don’t get where the table comes from
Explanation	I don’t see what you’re comparing, isn’t the urine analysis the same as the urine culture? So then I don’t see what are you comparing exactly	Did you make it and is it in your CAT or is it from the paper by Lee et al.?
Suggestion	So maybe you can be more specific in describing what you’re comparing?	I think you can be more specific about the source of your data

After the text with annotated comments had been written, the first author (FvB) used this text with the annotated comments to write a video script for the peer model. In this script, the model explained which parts of the text she reviewed and which thoughts came to her mind while she was reviewing the text. The script also described which comments she wrote in the paper. The script was used to record a video in which a professional actress played the peer model. This video was filmed and edited by a video expert and approximately 7 min in length. The video contained a split screen, one showing the actress while she was reviewing the text and the other showing the computer screen as she was typing her feedback. On that computer screen, the students’ paper was shown in the same electronic learning environment (ELO) that students worked in. The feedback comments that the actress wrote appeared on the screen while she was typing them.

The communication teachers received a lesson plan and PowerPoint slides for the feedback training. The lesson plan was the same for all teachers with the exception of the experimental treatment (MD, NM, or M). The first author (FvB) discussed the lesson plan individually with each teacher without stating the research hypothesis. The lesson plan started with the teacher explaining Hattie and Timperley’s (2007) three principles of feedback: feed-up, feed-back and feed-forward. To illustrate feed-up, the teacher showed a part of the scoring rubric that would be used to assess students’ CAT-paper. The teacher then explained the good feedback principles, i.e. that feedback should contain an evaluation, an explanation for the evaluation and a suggestion for improvement [42]. The teachers then gave additional tips to give positive feedback and points for improvement, to focus on helping and to give concrete suggestions or solutions. The remainder of the lesson plan was specific for each experimental treatment and will be explained under ‘Study procedure’.

### Study procedure

**Fig. 1:**
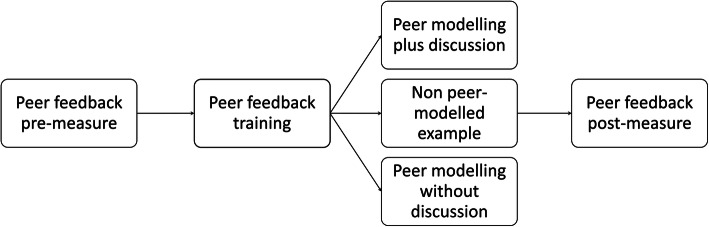
Overview of the experimental procedure.

A schematic overview of the experimental procedure can be found in Fig. 1. Before the training, students were instructed to write the first chapter of their CAT-paper and to upload this chapter in the ELO. Within the ELO, each student was assigned randomly and non-anonymously to two other students for peer review. Thus, each student performed and received two peer reviews. Students gave peer feedback in the ELO by writing annotated comments in their peers’ draft papers. They could consult an assessment rubric in the ELO, but were not required to use the rubric to give feedback.

After completing the peer reviews on the first chapter, students were instructed to upload the second chapter of their CAT-paper into the ELO and to bring their laptop to a subsequent workgroup session. These workgroups were assigned randomly to the three experimental conditions: peer modelling plus discussion (MD), non-peer modelled example (NM) and peer modelling without discussion (M). In the MD-condition, students observed the video of the peer model and the teachers paused the video three times to discuss the modelled feedback principles (i.e. evaluation, explanation for evaluation and suggestions for improvement). Students were instructed to identify these principles in the feedback and to discuss why these were good feedback principles. The M-condition followed the same procedure, except that the video was not paused for peer discussion. In the NM-condition, students did not observe the video, but instead read a hand-out of the paper with the annotated feedback comments. This was the same paper with the same feedback comments as in the video. Reading the feedback comments was followed by the same discussion as in the MD-condition, i.e. students identified the good feedback principles in the comments and discussed why these were good feedback principles.

After these experimental treatments, all students were instructed to peer review one of their peers’ second chapters on their laptop by writing annotated comments. They worked in silence and were instructed to complete both their peer reviews after the workgroup session.

### Analyses of the peer feedback

The peer feedback comments that students wrote before and after the training served as a pre- and post-measure of peer feedback. The feedback comments were coded based on an existing framework for coding peer feedback [42]. In this framework, four *functions* of feedback are distinguished: analysis, evaluation, explanation and revision. ‘Analysis’ means feedback aimed at understanding the text, whereas ‘evaluation’ refers to implicit and explicit quality judgments. ‘Explanation’ contains arguments that support an evaluation and ‘revision’ means suggestions for improvement. For ‘evaluation’, we further specified *positive evaluation* and *negative evaluation* and for ‘explanation’, we specified *explanation for evaluation* and *explanation for revision*. The feedback coding framework [42] also distinguishes three *aspects* of feedback: *content*, *structure* and *style*. ‘Content’ refers to feedback on the content of the text, e.g. its’ relevance, argumentation and clarity. ‘Structure’ refers to the internal consistency of the text and ‘style’ to the use of language, grammar and spelling. Thus, the eventual coding framework contained nine measures of peer feedback: six functions and three aspects.

The first author (FvB) and a second coder, who was unaware of the research hypothesis, independently coded the functions and aspects of four randomly selected students in iterative rounds of coding. They coded the functions and aspects of one student, compared their results, discussed differences in interpretations and made a list of coding agreements. Subsequently, they coded the functions and aspects of the next student, etcetera. After the fourth student, they had reached a satisfactory inter-observer agreement (Cohen’s Kappa = 0.87). Subsequently, the second coder coded the remainder of the peer feedback.

Following Field [43], assumptions of normality were checked with tests of normality (Kolmogorov-Smirnov) and by looking at the frequency distributions of the coded peer feedback. The Kolmogorov-Smirnov tests were performed on the nine post-measures within each experimental condition. This resulted in 27 tests (9 post-measures times 3 condition). 23 of these tests revealed significant p-values (*p* = .04 or lower). Four tests were non-significant: negative evaluation in the M-condition, *D*(59) = 0.09, *p* = .20, explanation for revision in the M-condition, *D*(59) = 0.10, *p* = 20, content in the MD-condition, *D*(36) = 0.12, *p* = .20 and style in the NM-condition, *D*(46) = 0.11, *p* = .19. Visual inspection revealed a positive skew (i.e. skew to the right) for all the dependent variables. This confirmed that the data was non-normally distributed. Therefore, non-parametrical Kruskall-Wallis tests were used to check for significant between-group differences in the pre- and post-measures of peer feedback.

## Results

We obtained complete feedback data of 141 participants (MD-condition: *n* = 36, NM-condition: *n* = 46; M-condition: *n* = 59). Table 2 (pre-measure) and Table 3 (post-measure) give an overview of all the different types of peer feedback that students gave. As can be seen in Table 3, the most prevalent type of feedback after the intervention was revision of style (e.g. ‘Maybe you could remove one or two commas out of this sentence, so it might have a smoother flow’). This type of feedback occurred 1545 times, covering 27.18 % of the total provided peer feedback. The next most frequently occurring feedback was analysis of content (e.g. ‘What is Optiflow therapy?’), which occurred 754 times (13.26 % of the total feedback). The third most frequently provided type of feedback was revision of content (e.g. ‘Maybe you can explain what reduction actually entails’). This type of feedback was given 702 times, or 12.35 % of the total feedback. Together, these three types of feedback contained more than half of the peer feedback. Thus, students commented predominantly on style-issues and clarification of the text.

**Table 2:** Frequencies of the pre-measure peer feedback functions and aspects

	Function							
**Aspect**		Positive evaluation	Negative evaluation	Explanation for evaluation	Revision	Explanation for revision	Analysis	**Total**
	Content	400	130	50	489	140	462	1671
	Structure	76	20	11	120	60	1	288
	Style	65	204	28	2074	370	104	2845
	**Total**	541	354	89	2683	570	567	4804

Note: The frequencies represent the total number of feedback functions and aspects across the three experimental conditions (*N* = 141)

**Table 3:** Frequencies of the post-measure peer feedback functions and aspects

	Function							
**Aspect**		Positive evaluation	Negative evaluation	Explanation for evaluation	Revision	Explanation for revision	Analysis	**Total**
	Content	583	330	102	702	328	754	2799
	Structure	49	30	21	151	70	8	329
	Style	94	237	46	1545	542	93	2557
	**Total**	726	597	169	2398	940	855	5685

Note: The frequencies represent the total number of feedback functions and aspects across the three experimental conditions (*N* = 141)

The Kruskal-Wallis tests (see Table 4) revealed no significant between-group differences in peer feedback before the feedback training. In contrast, there was one significant difference in peer feedback after the training: students in the MD-condition gave significantly more positive feedback than students in the NM-condition, *H*(2) = 6.33, *p* = .04. Pairwise comparisons corrected for multiple comparisons (Bonferroni) showed a higher degree of positive evaluation in the MD-condition than in the NM-condition, *p* = .04. There were no significant differences in positive evaluation between the NM-condition and the M-condition, *p* = .62 and between the MD-condition and the M-condition, *p* = .43. Table 5 shows the median feedback scores on the post-measure, including the inter quartile ranges (IQRs). As can be seen in that table, the IQRs were large, meaning the amount of provided peer feedback varied substantially between students.

**Table 4:** Kruskal-Wallis tests of the peer feedback pre- and post-measures

	Pre-intervention	Post-intervention
**Functions**		
Positive evaluation	*H*(2) = 0.75, *p* = .69	* H*(2) = 6.33, *p* = .04*
Negative evaluation	*H*(2) = 0.02, *p* = .99	* H*(2) = 2.21, *p* = .33
Explanation for evaluation	*H*(2) = 1.08, *p* = .58	* H*(2) = 5.25, *p* = .07
Revision	*H*(2) = 5.72, *p* = .06	* H*(2) = 1.25, *p* = .54
Explanation for revision	*H*(2) = 4.24, *p* = .12	* H*(2) = 0.63, *p* = .73
Analysis	*H*(2) = 1.08, *p* = .58	* H*(2) = 4.51, *p* = .11
**Aspects**		
Content	*H*(2) = 1.36, *p* = .51	* H*(2) = 4.48, *p* = .11
Structure	*H*(2) = 0.29, *p* = .87	* H*(2) = 0.07, *p* = .97
Style	*H*(2) = 0.4.30, *p* = .12	* H*(2) = 0.31, *p* =. 86

* Significant at *p* < .05

**Table 5:** Median post-measure peer feedback scores per experimental condition

	MD (*n* = 36)	NM (*n* = 46)	M (*n* = 59)
	*Median (IQR)*	*Median (IQR)*	*Median (IQR)*
**Functions**			
Positive evaluation	7.50 (16)*	4.00 (6)*	*6.00 (8)*
Negative evaluation	*3.00 (5)*	*3.00 (6)*	*2.00 (4)*
Explanation for evaluation	*1.00 (3)*	.*00 (3)*	.*00 (1)*
Revision	*11.50 (16)*	*12.00 (13)*	*14.00 (15)*
Explanation for revision	*5.00 (7)*	*6.00 (6)*	*5.00 (7)*
Analysis	*4.00 (9)*	*3.00 (5)*	*3.00 (9)*
**Aspects**			
Content	*22.50 (34)*	*12.50 (12)*	*17.00 (20)*
Structure	*1.00 (4)*	*1.00 (4)*	*1.00 (4)*
Style	*8.50 (15)*	*10.00 (13)*	*12.00 (17)*

MD = peer modelling plus discussion; NM = non-peer modelled example; M = peer modelling without discussion; IQR = Interquartile range

* Significant difference (*p* < .05)

## Discussion

Teaching and communicating clearly are important physician skills [1, 2]. Students can practice these skills by giving peer feedback. Therefore, we developed a training in which a peer model demonstrated on video how to give peer feedback, after which students discussed the good feedback principles that she modelled. Since peer discussion can enhance cognitive elaboration [34, 35], we expected peer modelling plus discussion of the modelled feedback principles to have a beneficial effect on students’ use of the feedback principles compared to (1) only observing the peer model or (2) discussing the feedback principles without observing the peer model. However, except for positive evaluation, we found no significant differences between the three conditions in the amount of peer feedback that students gave. Positive evaluation was provided significantly more often in the MD-condition than in the NM-condition.

A reason for the lack of significant effects was that the amount of peer feedback varied a lot between students, suggesting that some students put considerably more effort into giving feedback than others. This notion can be supported by a previous study in which students experienced large differences in quality of peer feedback they provided and received. Students reported giving more peer feedback than they received, delays in receiving peer feedback until the day of the submission deadline, and hearing from other students that they were too busy to give peer feedback. Students also reported that peers should be held accountable for giving good feedback. Moreover, students who were committed to giving good feedback seemed to be driven by ‘academic altruism’, an altruistic motivation to provide good peer feedback [44].

It is also possible that students’ motivation to give peer feedback varied depending on how much they valued the peer feedback task. According to expectancy-value theory, the perceived utility value of a task promotes engagement with that task [45]. This mechanism may also apply in contexts in which students give peer feedback [46]. That is, engagement with peer feedback may depend on how important students find peer feedback for developing their own skills. A high perceived utility value has been found to be positively associated with performance [47]. Therefore, students who value the utility of giving peer feedback may also give better feedback. This may explain why some students gave more and better feedback than others.

The finding that the MD-condition gave significantly more positive feedback than the NM-condition may be explained by the fact that the peer model demonstrated explicitly how to give positive feedback and that students elaborated on this principle in the MD-condition. Students in the NM-condition did not see the demonstration of positive feedback, although they could read it in the hand-out. We should also remind the reader here that the teachers encouraged giving positive feedback. Therefore, this seemed to be more evident than other aspects that were modelled. The possible effect of modelling on positive feedback can be seen as a desirable outcome, as feedback should encourage motivation and self-esteem [48, 49] under the condition that it is aimed at the task and not the person [50].

Our findings show that students provided more positive than negative peer feedback in general, which is in line with previous studies [23, 51]. Students may feel uncomfortable in giving negative peer feedback because they wish to maintain good social relationships with their peers [14]. Although this seems understandable, it has also been argued that ‘balancing rules’ for positive and negative feedback, such as the sandwich method, can harm the authenticity of feedback processes and put too much focus on feedback messages instead of using feedback for improvement [52]. Therefore, although positive feedback should be encouraged, the added value of ‘balancing rules’ can be debated.

Although the purpose of this study was not to compare pre- and post-intervention frequencies of peer feedback, these frequencies do perhaps provide useful information for designing peer feedback training. In both the pre- and post-measure, the most occurring type of feedback was revision on style. In general, revision on style consisted of simple, local (i.e. sentence-level) suggestions for revision. However, high quality peer review is characterised by a balanced mix of feedback on both global and local text issues [20]. Therefore, educators may focus peer feedback training more on providing global-level feedback in order to improve the quality of peer feedback.

A question that can be raised is whether the outcomes would have been different in a different learning task. Providing feedback on academic writing requires different skills than on clinical tasks. For instance, peer review requires the skill to distinguish global and local writing issues [20] and detect problems in style, structure and content of writing [42]. As a comparison, feedback on (for instance) clinical consultation may require the skill of detecting problems in building a trusting relationship with the patient, structuring a consultation and dealing with patients’ emotions [53]. These are very different skills.

Also, students may attribute more utility value to learning tasks that they perceive as more clinically or professionally relevant than academic writing. This seems to be an unresearched area. To our knowledge, the criteria ‘evaluation’, ‘explanation’ and ‘suggestion’ (or similar criteria) have only been used to analyse written peer feedback on academic writing [54–57] or concept maps [58]. Therefore, we also applied the feedback training to a writing task.

However, to our knowledge there is no research examining the quality of peer feedback provided on other tasks than written tasks. For instance, one study investigated the effect of expert- and peer feedback on ratings of students’ communications skills, but not the peer feedback that students provided [11]. In a review study of peer feedback during collaborative learning, no studies were found in which faculty evaluated the quality of feedback [9]. Also in a recent scoping review on feedback for early career professionals, no findings on feedback quality are reported [59]. Therefore, it seems that peer feedback has not been analysed in learning tasks other than written tasks, although the criteria that can be used to analyse feedback seem to be generic [25, 40–42]. This may call for new research to peer feedback in other learning tasks as well, especially in professional learning tasks that students find relevant.

### Limitations

There are some limitations to this study. First, not all students who gave informed consent fully completed the peer feedback assignment. A possible reason for this is that students gave peer feedback at later time points, outside of the approved ELO. Still, it should be noted that the majority of the students did give peer feedback in the ELO.

Second, due to the online setting of the peer feedback process, face-to-face feedback dialogue between peers was not possible. Feedback dialogue prevents a unilateral transmission of feedback from the provider to the receiver and can therefore lead to a better shared understanding of the feedback and facilitate acting upon the feedback [60, 61]. However, research shows that students do not frequently engage in online feedback dialogue after giving and receiving peer feedback [62]. Therefore, a future challenge for online peer feedback may be to promote online feedback dialogue between students.

Third, we did not collect feedback from the students on the training and on their perceptions of feedback in general. This would have provided more insights in why the present results were found. Future research should provide more explanatory evidence on why certain effects do or do not occur.

## Conclusions

Although modelling good feedback principles and discussing these modelled principles may increase students’ use of positive feedback, students also differ substantially in the amount of peer feedback that they give each other. This suggests that additional incentives are needed to motivate students to give peer feedback. Such an incentive could be to provide feedback training in learning tasks that students perhaps see as more clinically relevant. A further incentive may be to emphasise the utility value of peer feedback as preparation for future teaching practice. Another incentive could be to stress the importance of altruism and professional accountability in the peer feedback process. Future interventions may focus on such incentives in order to encourage high-quality peer feedback. Perhaps the next step is to add an explanation of the importance and relevance of feedback training and apply the feedback training model to a clinical subject.

## Data Availability

The anonymised dataset and SPSS-syntax can be sent by the corresponding author upon reasonable request.

## Abbreviations

CAT: Critical Appraisal of a Topic
ELO: Electronic Learning Environment
M: Peer modelling without discussion
MD: Peer modelling plus discussion
NM: Non-peer modelled example
